# Clinical value of endoscopic ultrasound sound speed in differential diagnosis of pancreatic solid lesion and prognosis of pancreatic cancer

**DOI:** 10.1002/cam4.7026

**Published:** 2024-03-13

**Authors:** Jianing Qiu, Kangrong Li, Xiuyan Long, Xiaoyu Yu, Pan Gong, Yu Long, Xiaoyan Wang, Li Tian

**Affiliations:** ^1^ Department of Gastroenterology The Third Xiangya Hospital of Central South University Changsha China; ^2^ Department of Pediatrics The Third Xiangya Hospital of Central South University Changsha China; ^3^ Health Management Center of the Third Xiangya Hospital of Central South University Changsha China

**Keywords:** endoscopic ultrasonography, pancreatic cancer, pancreatic solid lesions, sound speed

## Abstract

**Background:**

Differential diagnosis of pancreatic solid lesion (PSL) and prognosis of pancreatic cancer (PC) is a clinical challenge. We aimed to explore the differential diagnostic value of sound speed (SS) obtained from endoscopic ultrasonography (EUS) in PSL and the prognostic value of SS in PC.

**Methods:**

Patients with PSL in The Third Xiangya Hospital of Central South University from March 2019 to October 2019 were prospectively enrolled, who obtained SS from PSL. Patients were divided into the PC group and the pancreatic benign lesion (PBL) group. SS1 is the SS of lesions and SS2 is the SS of normal tissues adjacent to lesions. Ratio1 is equal to SS1 divided by SS2 of PSL (ratio1 = SS1/SS2).

**Results:**

Eighty patients were enrolled (24 PBL patients, 56 PC patients). SS1 and ratio1 in PC group were higher compared with PBL group (SS1:1568.00 vs. 1550.00, *Z* = −2.066, *p* = 0.039; ratio1: 1.0110 vs. 1.0051, *Z* = −3.391, *p* = 0.001). The SS1 in PC (*Z* = −6.503, *p* < 0.001) was higher compared to SS2. In the nonsurgical group of PC, low ratio1 predicted high overall survival (OS) (7.000 months vs. 4.000 months; *p* = 0.039). In the surgical group of PC, low SS1 was associated with low median OS (4.000 months vs. 12.000 months; *p* = 0.033).

**Conclusions:**

SS plays a vital role in distinguishing between PBL and PC. Higher SS1 and ratio1 obtained by EUS are more related to PC than PBL. In PC patients, high SS1 may predict pancreatic lesions. In the nonsurgical group of PC, low ratio1 may predict high OS. However, in the surgical group of PC, low SS1 may predict low OS.

## 1 BACKGROUND

Pancreatic solid lesions (PSLs) are divided into neoplastic lesions and pancreatic benign lesions (PBLs): neoplastic lesions are primarily pancreatic ductal adenocarcinoma, which accounts for 70% to 95% of PC^1^; PBLs include chronic pancreatitis, autoimmune pancreatitis, and paraduodenal pancreatitis.^2^ A lack of effective early screening methods and the asymptomatic or atypical nature of PC in its early stages are reasons that may cause its low early diagnosis rate and poor prognosis. The 5‐year survival rate for PC is less than 9%.^3^ However, there exists a challenge in differentiating benign and malignant PSL.^4^ Currently, PC is early diagnosed primarily using imaging, and related studies have demonstrated that multidetector computed tomography (MDCT) and MRI have diagnostic sensitivity of 76%.^5^ In terms of clinical symptoms and imaging, PC and chronic pancreatitis with mass have many similarities, and 5% to 35% of mass‐forming chronic pancreatitis were misdiagnosed as PC, and then pancreaticoduodenectomy was performed.^6^ Moreover, if PC is not detected and accurately diagnosed in time, they may progress and treatment can be delayed, resulting in serious health consequences.

Endoscopic ultrasonography (EUS) has a higher diagnostic rate for pancreatic diseases than conventional imaging methods such as CT and MRI,^7^ and it is currently considered as one of the most accurate method for diagnosing pancreatic diseases. Even though endoscopic ultrasound‐guided fine needle aspiration/fine needle biopsy (EUS‐FNA/FNB) can obtain lesion tissues for a clear diagnosis, its diagnostic rate is only 80% to 95%, which does not meet the clinical requirements.^8^ Additionally, EUS‐FNA/FNB is an invasive procedure with risks of bleeding, pancreatitis, and tumor implantation.^9^ EUS elastography has limited clinical application due to its poor reproducibility and low negative predictive value.^10^ Ultrasonography may show false‐positive contrast signals in non‐vascularized areas of pancreatic masses. At the same time, excessive contrast doses may also cause signal attenuation, and its differential diagnostic rate for benign and malignant pancreatic masses is about 77% to 91%.^11^, ^12^ In contrast, sound speed (SS) of EUS is a new noninvasive method for virtual touch tissue quantification, which does not rely on physicians' experience and has the advantages of objectivity, convenience and noninvasiveness. Only one research shows that SS can be applied to EUS‐guided tissue collection of pancreatic masses,^13^ suggesting that SS has a very promising application in noninvasive adjunctive diagnosis.

As for the prognosis of PC, patients' surgical margin status, tumor grade, presence of lymphatic infiltration, preoperative and postoperative serum CA19–9 levels, and smoking status^14^, ^15^, ^16^ all influence it. However, it remains controversial whether these indicators can be used to predict the prognosis of PC.^17^ According to studies conducted with EUS elastography,^18^ patients with higher strain rates have a poorer prognosis. This study aims to investigate whether SS can differentially diagnose PSL and whether SS can be used as a valid prognostic indicator for PC.

## 2 METHOD

### 2.1 Patient

Patients with PSL receiving endoscopic ultrasonography‐sound speed (EUS‐SS) measurement in the Third Xiangya Hospital of Central South University between March 2019 and October 2019 were involved. Subjects with one or more of the following were excluded: age <18 years, nonsolid pancreatic lesions revealed by imaging examination, severe cardiovascular disease, high risk for profound sedation, allergy to anesthetics, acute pancreatitis in the previous 2 weeks, pregnancy or lactation, any reason leading to unreliable follow‐up or absence of informed consent. The check on relevant imaging examinations was carried out before performing EUS.

A total of 80 patients with PSL were diagnosed as PC or PBL according to results of surgical pathology or cytology/histology after EUS‐FNA/B or clinical outcomes at 8 months follow‐up. The overall survival was obtained by phone follow‐up.

This prospective trial was conducted in the endoscopic center of a tertiary hospital in China, approved by the Institutional Review Board of Third Xiangya Hospital, Central South University (received permission on January, 30, 2019, No. R19005). All patients signed informed consent forms regarding all procedures.

### 2.2 EUS‐SS and procedural technique

**FIGURE 1:**
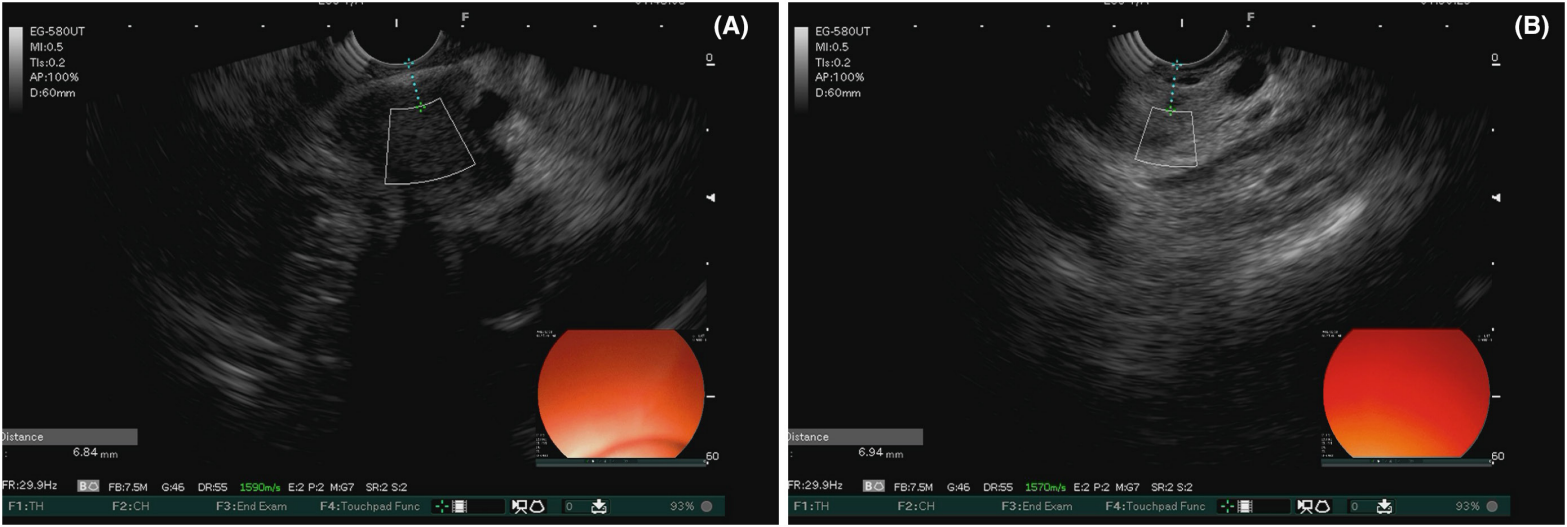
Sample graph of the SS as obtained by EUS.A SS of lesion area B SS of adjacent area.

All patients enrolled were subjected to a routine EUS examination (Fuji SU‐9000), and the SS correction measurement for the region of interest were conducted in compliance with ethical guidelines for obtaining patient consent, which was equipped with advanced image processing technology for estimating the optimal SS in the body so as to make SS set by the system consistent with the actual SS. The SS correction measured SS of the target tissue adopting SS of normal tissue (1540 m/s) as a reference to provide hardness information. The SS was measured at three random points of the lesion and in the adjacent area of the lesion respectively. A sample graph from the SS is presented in Figure 1. The averages of SS1 and SS2 were calculated. The EUS‐SS was completed by two experienced endoscopic doctors.

In this study, for EUS‐FNA, we employed the Expect™ Slimline Endoscopic Ultrasound Aspiration Needle (Boston Scientific Corporation, Natick MA, USA). On the other hand, EUS‐FNB was performed using the Cook EchoTip ProCore needle. Both EUS‐FNA and EUS‐FNB procedures utilized the modified wet suction technique. For punctures in the head, neck, and body of the pancreas, a 22G needle was chosen, while a 19G needle was used for the tail of the pancreas.

## 3 STATISTICAL ANALYSIS

Statistical analyses were performed using SPSS (version 22.0; IBM, Armonk, NY, USA). In determining our study's sample size, we employed a 2‐sided hypothesis test with a significance level set at *α* = 0.05 and a power of 80%. Based on these criteria, a cohort of 80 patients was deemed necessary to achieve the desired statistical rigor for our research objectives. Descriptive statistics were summarized for all demographic data covering age, sex, body mass index (BMI), SS1, site of lesions in the pancreas and lesion size. Continuous variables were represented as Mean ± SD and categorical variables were reported as frequency and percentage. The SS1 and ratio1 between PBL and PC were compared by a Wilcoxon rank sum test, respectively. The comparison of the difference between SS1 and SS2 of each patient was conducted with the Wilcoxon signed‐rank test in PC group. Survival time was analyzed using Kaplan–Meier curves. A *p* < 0.05 was considered statistically significant.

## 4 RESULTS

In this prospective and single‐center study, a total of 80 patients (male: 45, female: 35, mean age: 59.4 ± 11.7 years old) were enrolled. The PBL group involved 8 (33.3%) females and 16 (66.7%) males, with a mean age of 56.1 ± 10.8 years; the PC group involved 27 (48.2%) females and 29 (51.8%) males, with a mean age of 60.7 ± 11.6 years. According to the pathological results, 20 (83.3%) were categorized as pancreatitis, 2 (8.33%) with autoimmune pancreatitis and 2 (8.33%) with tuberculosis in PBL group; 55 (98.2%) with pancreatic ductal adenocarcinoma and 1 (1.80%) with neuroendocrine tumors in PC group. The demographic data are summarized in Table 1.

**TABLE 1:** Clinical characteristic of included cases.

Characteristic	Benign lesion group *n* = 24	Malignant lesion group *n* = 56	*p* value/Z value
Age (years), mean ± SD	56.1 ± 10.8	60.7 ± 11.6	0.11
Sex, *n* (%)			
Male	16 (66.7%)	29 (51.8%)	0.163
Female	8 (33.3%)	27 (48.2%)	
BMI, mean ± SD	21.2 ± 3.1	20.4 ± 2.8	0.246
SS1 (m/s), mean ± SD	1560.2 ± 21.0	1568.1 ± 12.3	0.039
Site of lesions in the pancreas, *n* (%)			
Ampulla and head and neck	18 (75.0%)	33(58.9%)	0.132
Body and tail	6 (25.0%)	23 (41.1%)	
Lesion size, *n* (%)			
<3 cm	19 (79.2%)	26 (46.4%)	0.006
≥3 cm	5 (20.8%)	30 (53.6%)	
Final diagnosis, *n* (%)			
Pancreatitis	20 (83.3%)	‐	
Autoimmune pancreatitis	2 (8.33%)	‐	
Tuberculosis	2 (8.33%)	‐	
Pancreatic ductal adenocarcinoma	‐	55 (98.2%)	
Neuroendocrine tumor	‐	1 (1.8%)	
Anticancer treatment			
Surgery	‐	14 (25.0%)	
Chemotherapy	‐	12 (21.4%)	
Conservative management	‐	24 (42.9%)	
Surgery + chemotherapy	‐	6 (10.7%)	

*Note*: All of the continuous variables except for SS1 of benign lesion group and BMI of malignant lesion group followed normal standard distribution. SS1 and BMI was compared by Wilcoxon rank sum test.

### 4.1 The value of SS on the identification of lesions

**FIGURE 2:**
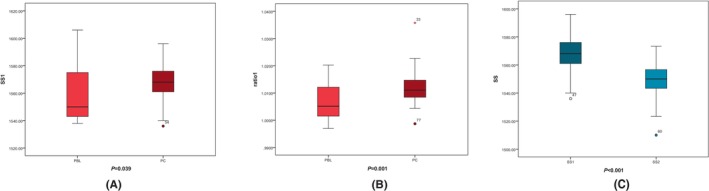
The value of SS on identification of the lesions. (A) The Wilcoxon rank sum test was adopted to compare the SS1 between PBL and PC (B) The Wilcoxon rank sum test was adopted to compare the ratio1 between PBL and PC (C) The Wilcoxon signed‐rank test was adopted to compare the SS1 and SS2 in PC patients.

As the absence of normal distribution for SS1 of PBL group and ratio1 of PC group, a Wilcoxon rank sum test was adopted to compare the SS1 and ratio1 between PBL group and PC group. A significant difference was observed in the SS1 and ratio1 that all of them in PC group are higher in median value compared with PBL group (SS1:1568.00 vs. 1550.00, *Z* = −2.066, *p* = 0.039; ratio1: 1.0110 vs. 1.0051, *Z* = −3.391, *p* = 0.001). The details of SS1 and ratio1 in the PBL and PC group are shown in Figure 2A,B. This result indicated that EUS to measure SS1 and ratio1 was an excellent noninvasive method for distinguishing malignancy of PSL.

The difference between SS1 and SS2 of PC group was further explored. The SS1 and SS2 of every patient were compared by adopting the Wilcoxon signed‐rank test for PC group. The median value of SS1 is higher than SS2 (1568.00 vs. 1550.00) in PC group. The mean SS significantly varies between lesion area and adjacent area in PC patients (*Z* = −6.503, *p* < 0.001), indicating the role of high SS1 in targeting lesion sites. The details of SS1 and SS2 in the PC group are shown in Figure 2C.

### 4.2 Prognosis value of SS for PC

To further investigate the prognosis value of SS1 and ratio1 for PC, the PC group was first divided into a surgical group and a nonsurgical group.

In the nonsurgical group, the median value of 1568 m/s for SS1 (1548–1592 m/s) was set as the cutoff value of SS1, based on which the patients were divided into high SS1 and low SS1 group, 18 in high SS1 group and 18 in low SS1 group. The median ratio1 was 1.0118 (0.9987–1.0358). We set 1.012 as the cutoff value of ratio1, according to which the patients were divided into high ratio1 and low ratio1 group, 17 in high ratio1 group and 19 in low ratio1 group.

**FIGURE 3:**
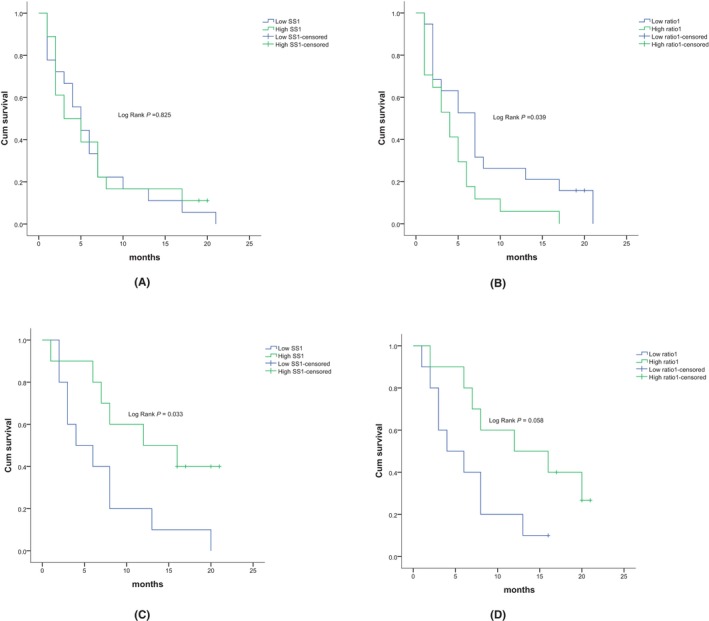
Prognosis value of SS1 and ratio1 in non‐surgical group and surgical group of PC. (A and B) correspond to the non‐surgical group, while (C and D) correspond to the surgical group (A) This is a survival curve of low SS group and high SS group of nonsurgical patients, with the horizontal coordinate being the survival time after the diagnosis of a malignant lesion. The level of SS1 had no suggestive significance for survival in nonsurgical group. (B) This is a survival curve of low ratio1 group and high ratio1 group of nonsurgical patients, with the horizontal coordinate being the survival time after the diagnosis of a malignant lesion. Low ratio1 was relevant to long survival time. (C) This is a survival curve of low SS group and high SS group of the surgical group, with the horizontal coordinate being the survival time after the diagnosis of a malignant lesion. Low SS1 was associated with short median survival time. (D) This is a survival curve of low ratio1 group and high ratio1 group of the surgical group, with the horizontal coordinate being the survival time after the diagnosis of a malignant lesion. The level of ratio1 had no suggestive significance for survival.

The Kaplan–Meier analysis was used to evaluate the prognostic significance of mean SS1 and ratio1, respectively. Low SS1 was associated with high median OS (5.000 months vs. 3.000 months; *p* = 0.825; Figure 3A), and low ratio1 was relevant to high median OS (7.000 months vs. 4.000 months; *p* = 0.039; Figure 3B).

In the surgical group, the median value of 1565 m/s for SS1 (1536–1596 m/s) was set as the cutoff value of SS1, based on which the patients were divided into high SS1 and low SS1 group, 10 in high SS1 group and 10 in low SS1 group. The median of ratio 1 was 1.0088 (1.0044–1.0209). We set 1.009 as the cutoff value of ratio1, according to which the patients were divided into high ratio1 and low ratio1 group, 10 in high ratio1 group and 10 in low ratio1 group.

The Kaplan–Meier analysis was used to evaluate the prognostic significance of mean SS1 and ratio1, respectively. Low SS1 was associated with low median OS (4.000 months vs. 12.000 months; *p* = 0.033; Figure 3C) and low ratio1 related to low median OS (4.000 months vs. 12.000 months; *p* = 0.058; Figure 3D).

## 5 DISCUSSION

An accurate differential diagnosis of PBL from PC still remains challenging.^4^ The current difficulty points to the possibility of certain PBL acting the same as PC at imaging, which increases the preoperative misdiagnosis rate and may result in unnecessary surgery.^6^ The traditional diagnostic methods of PC refer to imaging examinations, mainly involving MRI, MDCT, and EUS.^19^ In comparison to CT and MRI, EUS is more sensitive in the detection of PC, which can be used for early diagnosis. EUS is advantageous in detecting pancreatic masses, especially small PC, but fails to reach 100% sensitivity and specificity.^20^ Despite the high sensitivity and specification of EUS‐FNA/FNB in detecting PC,^21^, ^22^, ^23^, ^24^ it still produces false‐negative results,^25^ not to mention its invasion property. Therefore, a noninvasive, safe and inexpensive differential diagnosis method is still worth exploring. The SS mode of EUS serves as a noninvasive method that provides quantification of tissue hardness based on SS without the requirement of unique materials or preparation, as well as the reliance on experienced doctors. SS can objectively and precisely measure the distance and invasion of colorectal tumors.^26^ It can also reflect the hardness of the lump. There has been no clinical trial on SS in PSL.

This study initially investigated the value of SS measured under EUS for the differentiation of benign and malignant PSL. To our knowledge, this is the first study in the appliance of SS measurement on distinguishing PC and PBL. We tried to determine an appropriate way to assess benign and malignant lesions of PSL by SS, so two indicators (SS1 and ratio1) were calculated. These two indicators were consistent in determining the benignity and malignancy of PSL that was all two indicators of PC were higher than PBL, so the combination of these two values could be a noninvasive way to jointly assess the benignity and malignancy of PSL. Based on the median value of this cohort, it was speculated that PSL with SS1 greater than the median value of 1568 or ratio1 greater than 1.0110 might be malignant. In a previous study, SS depends on the degree of tissue fibrosis in the liver.^28^ Therefore, authors can reasonably extrapolate that SS of pancreatic tissue also correlates with the degree of fibrosis. However, PC and chronic pancreatitis can cause fibrosis of the pancreatic tissue.^29^ Therefore, it is not certain whether the differential diagnostic role of SS correlates with the degree of fibrosis. What's more, Boozari et al. speculated that adipose tissue interfered with SS. So it may also be necessary to design models for the calculation of SS that remove the interference of adipose tissue. In another study, Hirooka et al. reported no different SS provided by automatic adjustment of SS according to pancreatic cystic lesions and solid lesions.^27^ It may be more advantageous to use other imaging modalities to differentiate between solid and cystic pancreatic lesions before performing SS.

For patients with suspected malignant lesions requiring biopsy, the precise targeting of the puncture site becomes a topic of interest. Despite the high accuracy of EUS‐FNA and EUS‐FNB (Accuracy of EUS‐FNA vs. EUS‐FNB: 87% vs. 78%), they fail to achieve the entire accuracy^30^ in diagnosing pancreatic malignancy. The pursuit of new methods to optimize targeting biopsy techniques remains a concern for clinicians. Contrast enhance‐endoscopic ultrasonography (CE‐EUS) and EUS elastography can act as an adjunct to EUS‐FNA by providing information around lesions^21^ but still fail to meet clinical requirements for sensitivity and specificity.^31^, ^32^ The analysis of 56 cases in this study indicates the significant difference in SS between lesion area and adjacent area in PC. The high SS area is more likely to be pathological tissue. SS measurement as a noninvasive method requires no unique materials or preparations. A previous case demonstrated that SS could elevate the diagnostic accuracy of EUS‐FNA by determining the optimal insertion location for fine‐needle aspiration, thus achieving an improvement in the diagnosis of PC.^33^ Although not all of our patients underwent EUS‐guided puncture, it can also be assumed that SS has a role in a targeted puncture in combination with our results and that case.

In recent years, researchers have investigated elastography in EUS to assess the strain ratio of PC^18^ and contrast‐enhanced harmonic EUS to assess the vascularity of PC^34^ to predict the succeeding development of PC patients. They have found the potential of EUS in assessing PC prognosis. What's more, it is known that high SS1 represents a malignant pancreatic lesion from the previous study section. Therefore, the authors tried to explore whether SS could also be a means for EUS to predict the subsequent development of PC patients. PC patients in this study were divided into two subgroups, surgical group and nonsurgical group, and then performed Kaplan–Meier analysis separately.

Our study provides valuable insights into the predictive role of SS for OS in PC patients. Given the potential variability in SS across different individuals and pancreatic tissue locations, we have not only focused on SS1, which represents the SS of the lesion, but also on ratio1, calculated as SS1/SS2, to predict OS in PC patients. This approach was designed to mitigate the impact of individual differences. Understanding the profound impact of surgery on OS in PC patients,^35^, ^36^, ^37^ we divided them into surgical and nonsurgical groups. This allowed us to delve deeper into the relationship between SS and OS in these two distinct sets of patients. Our findings indicate that in the nonsurgical group, low ratio1 may predict high OS, while in the surgical group, low SS1 may predict low OS. It's worth noting that our prior results only demonstrated differences in SS1 and ratio1 between benign and malignant lesions, without exploring the relationship between SS and the degree of malignancy. Therefore, our analysis should be considered as indicative of a trend rather than conclusive evidence.

We speculate that the observed differences could be attributed to several factors. First, in the nonsurgical group, a low ratio1 suggests that the SS in the cancerous region is closer to that in the normal region. This leads us to hypothesize that the tissue structure of lesion may not differ significantly from normal tissue, resulting in a relatively longer OS. However, this hypothesis requires further investigation to explore the relationship between pancreatic cancer (PC) tissue pathology and SS. Second, in the surgical group, low SS1 might be suggestive of a reduced OS. However, it's essential to note that in the surgical group, OS in PC patients is shaped by a myriad of intertwined factors, including pathological staging, chemotherapy, underlying health conditions, and nutritional status.^38^, ^39^ Such intricacies could provide context to the potential link between low SS1 and OS outcomes. Our study, focusing solely on SS and the presence or absence of surgery, may not provide a comprehensive view. Lastly, we acknowledge that the sample size in our study, particularly in the surgical group, is limited, which may introduce bias. Future research with a larger sample size and prospective design is needed to clarify the impact of SS on OS in PC patients with or without surgery.

There are two shortcomings in this study. First, this study failed to report diagnostic tests and plot receiver operator characteristic curve of SS1 and ratio1 due to the limitation of the number of cases included in this study. In the future, it is worthwhile to determine reliable cutoff values of SS1 and ratio for PC and PBL through a multicenter study with a large sample to initially screen patients with suspicious malignant lesions for the need for invasive biopsy. Second, follow‐up treatments and pathological features of lesions vary from each patient. Due to the insufficient number of cases included in this study, the prognostic effect of SS was not studied by grouping pathological types or subsequent treatment modalities. However, it is suggestive for multi‐center studies with larger sample size which control for a single variable.

In summary, we predict that a high SS obtained by EUS is associated with lesions of PC. In addition, SS may be able to be adopted as a reference index to target biopsy because SS is higher at the lesion site in this study than in the surrounding normal tissue. In non‐surgical group of PC, low ratio1 may predict high OS. While in surgical group of PC, low SS1 may predict low OS. However, it still needs further clinical study and histopathological research to confirm these conclusions and explain the potential mechanism.

## AUTHOR CONTRIBUTIONS

**Jianing Qiu:** Methodology (equal); writing – original draft (equal); writing – review and editing (equal). **Kangrong Li:** Methodology (equal); writing – review and editing (equal). **Xiuyan Long:** Methodology (equal); project administration (equal); supervision (equal). **Xiaoyu Yu:** Supervision (equal). **Pan Gong:** Supervision (equal). **Yu Long:** Supervision (equal). **Xiaoyan Wang:** Project administration (equal); resources (equal); supervision (equal). **Li Tian:** Project administration (equal); resources (lead); supervision (lead).

## FUNDING INFORMATION

This work is supported by Research funded by Hunan Provincial Science and Technology Department (2020SK2013).

## CONFLICT OF INTEREST STATEMENT

The authors declare that they have no competing interests.

## ETHICS STATEMENT

Our study was approved by ‘The Third Xiangya Hospital of Central South University’ (K19027). All patients signed informed consent forms regarding all procedures.

## Data Availability

The datasets analyzed during the current study available from the corresponding author on reasonable request.
